# The Medical Profession of Ancient Times

**Published:** 1857-01

**Authors:** 


### NORTH AMERICAN

### MEDICO-CHIRURGICAL REVIEW.

**JANUARY, 1857.**

### Analytical and Critical Reviews.

ART. 1.—*The Medical Profession in Ancient Times; an Anniversary Discourse, delivered before the New York Academy of Medicine.* By JOHN WATSON, M.D., Surgeon of the New York Hospital. Published by order of the Academy. New York, 1856. Pp. 222, 8vo.

*Histoire de la Médecine depuis son Origine Jusqu'au XIXme Siècle.* Par P. V. RENOUARD, M.D. Paris, 1846. Vols. 2, pp. 468 and 524.

*History of Medicine from its Origin to the Nineteenth Century.* With an Appendix, containing a Philosophical and Historical Review of Medicine to the Present Time. By P. V. RENOUARD, M.D. Translated by C. G. COMEGYS, M.D. 1 Vol. 8vo. pp. 719.

*Recherches Historiques sur l'Exercise de la Médecine dans les Temples chez les Peuples de l'Antiquité, &c. &c.* Par L. P. AUGUSTE GAUTHIER, D.M.P. Paris et Lyon, 1844. Pp. 232, 12mo.

*Historical Inquiries into the Practice of Medicine in the Temples among the Ancients.* By L. P. AUGUSTUS GAUTHIER, M. D.

IT is something gained in these days of waning scholarship, when the designation of learned implies in the minds of many who ought to know better unfitness for the practical duties of professional life, that a writer like Dr. Watson is found among us to sustain the claims of learning, by showing, in his own person, how well and gracefully it is united to the active and successful discharge of every-day clinical practice, both in the wards of an hospital, and in the houses of his private patients. Although the annals of medicine abound in similar examples of this union, as has been shown in the pages of one of our immediate predecessors,* we hail, nevertheless, the appearance of Dr. Watson's "Anniversary Discourse," not only as a good omen, but as itself preluding and installing in the medical profession in this country, a restoration of humane letters to the place which they occupied within a period certainly not remote, and a conscious acknowledgment of their influence in enlightening and enlivening our every-day path, and, at the same time, in aiding us to make future investigations and discoveries. We learn from them, on the one hand, how and where the human mind has wandered from the right direction, and wasted its energies in unprofitable no less than wearisome trials, and in disquisitions either barren of results, or only productive of rank and noxious weeds; and, on the other hand, the actual state of the exact and demonstrative sciences, so that, when bent on further progress, we know the precise starting-point. All that has been said in favor of the study of history in general, holds good, with certain limitations, of the study of the history of medicine; and if ignorance of the first be deemed a cause of regret to the person who is thus deficient, and of reproach from others who are better informed, how much more regretable and reproachful is ignorance in the members of a profession, called "learned" and "liberal," of its annals, and of the causes which have sometimes accelerated, sometimes retarded, the advancement of medical science and literature? Descending from the history of the progress and revolutions in medicine to the details of individual life, what rich stores of instruction, what incitements to rival effort, what noble examples of self-denial and benevolence, are contained in the biographies of the physicians of bygone times! These are men and deeds for us to be proud of, and to make us feel that we belong to an order which can boast of greater antiquity and broader blazonry than any living descendants of the Percys or the Montmorencys. Can there then be dullards among us who would forego the privilege of such associations, by remaining in ignorance, and shutting their eyes on the pages of history, with all its lessons of wisdom, and it may be, at times, of warning also, in the self-sufficiency of mistaken independence and of exclusive reliance on personal experience, in place of their correcting its fallacies, and strengthening its conclusions when just, by the experience of the keen and conscientious and calm observers which, from age to age, for the last twenty-two centuries, has been put on record? Evading the real question, it is sometimes alleged, that a man may be learned and ingenious without his being a judicious and successful practitioner. This is undoubtedly true; but does it follow that a stupid and an illiterate man will practise with more success? We are supposing now that each has enjoyed the same opportunities of contemporary clinical observation and instruction. Whether we speak of physicians or of soldiers, the pro-

* Medical Examiner, April, 1854.

babilities of efficiency are, it can hardly be denied, on the side of him who has been thoroughly trained and armed, and who has acquired the coolness and self-possession which allow of his employing his weapons, or his armamentarium of instruments and medicines, as the case may be, at the critical moment. The well-read physician, whose mind has been matured by study, has the same advantage over the illiterate one, if we can suppose a connection between illiteracy and medicine, that a well-trained soldier has over a raw recruit. Some there are who have won renown in arms without their having passed through military schools, or having received instruction in engineering: but we have yet to read of a truly great commander, one famed for his strategetical skill, who engaged in military life in entire ignorance of the art of war, as taught and practised by those who preceded him. So, also, in our own profession, there have been men of great and original genius, who have accomplished wonders, with but little aid derived from education; but, in reply, we would remark, how small is the number of such persons, and how much the value of their discoveries or original views have been diminished by their want of methodical habits of thought and practised dialectics. We need not cite a more remarkable example of the correctness of this view of the subject than that of John Hunter; and, if we had time to explain ourselves, we would add the name of one at least of John Hunter's most eminent pupils.

In handling the subjects which are discussed in the works whose titles head this article, we believe that, within the space allowed us, we shall do the most service to our readers by giving first a brief outline of the contents of the volumes, and then endeavor to represent their scope and spirit after our own fashion, and, for the most part, in our own language; inferior as both may be to those of the authors whose company we shall continue to keep on the occasion. By this means we shall be able to preserve a more connected narrative, historical and descriptive, than if we were to give a series of extracts with the same view; the more especially as this latter course would lead us far beyond our prescribed limits.

Dr. Watson, in a short preface, tells his readers of the bibliographical stores which he has made use of in preparing his discourse. They are very numerous, and include much of what has been written on the subject in the classical and the modern languages, in the way both of direct history and commentary, and of collateral literature and criticism. If the list which he introduces should alarm by its ponderous character, we cannot but feel the more grateful for the labor, skill, and good taste which he has exercised in elaborating from such massive and apparently heterogeneous materials, a work so clear and perspicuous as the one before us. His volume consists of thirteen chapters, in which he traces the condition of medicine, the merits and status of medical teachers and writers, and the succession of medical schools, from the earliest organization of society down to the time of Paul of Ægina, through the intermediate periods of the early Greek or ante-Hippocratic, and of the Asclepiadæ, then of Hippocrates and his immediate successors, on to the Schools of Pergamus and of Alexandria, and those of Smyrna and Epidaurus. Next follows a notice of the Schools of Rome, from their origin to the rise of the Eclectics, occupying one section, while a succeeding one is taken up with an account of the later Methodists of the Roman School. Greek writers and teachers, not of the Roman School, but contemporary with it, receive their share of attention. Galen, as so great a name deserves, has a chapter to himself. After him are enumerated certain Latin medical writers. Chapter XII presents us with a notice of Greek medical writers and medical institutions subsequent to Galen. The thirteenth and final chapter is taken up with a consideration of the laws and customs of the Roman Empire in relation to the Profession.

It augurs well for the progress of sound and Hippocratic medicine,—which is, at the same time, the medicine of learning and of common sense,—in the West, that a work of the character of M. Renouard's History should find a translator and a publisher in Cincinnati. The science of medicine, and the social position of its legitimate cultivators and professors, have suffered in the West,—as, indeed, where have they not suffered,—from the impudence and greed of ignorant pretenders and empirics, who find it easier to concoct crude speculations, and frame certain formulas of easy remembrance, which they embody in a system, so called, and teach, forsooth, under corporate grants, than to devote themselves, in a spirit of becoming industry and perseverance, to the study of the various branches of legitimate medicine during the requisite period, and then to submit with patience to, often, a long period of probation, before their professional services are required by the community in which they have taken up their abode. Out of the present apparent chaos, or of "confusion worse confounded," order will, however, yet come; and legitimate medicine, resting on the basis of sound philosophy and pure ethics, will appear in all its fair proportions. But, to obtain this desirable result, members of the profession, everywhere, must aim at and work up to a higher standard than that which is now generally received. Ignorance and illiteracy must not be tolerated within professional limits; pert assumption must be rebuked in those who start up as teachers before they have passed through a reasonable noviciate as learners; and the consciences of our state governments must be quickened into some sensibility for the health and lives of the people, so that a stop may be put to the cruel facility of granting charters to every batch of importunate applicants who wish to engage in the speculation of getting up a medical school, no matter under what name, or what doctrine or fashion of practice its half-fledged professors propose to teach. As contributing to a correct view of existing evils and tending to pave the way for amendments, the general perusal of such works as those of Dr. Watson and of M. Renouard should be encouraged, both by the example and precept of all those who in their respective positions may be supposed to be influential. The translation of the latter work by Professor Comegys is only one, among others, of the commendable zeal with which he turns to good account, for the honor of the profession, his knowledge acquired by previous study and foreign travel.

M. Renouard divides his History of Medicine into three books, corresponding to as many ages; and these again are subdivided into sections, or periods. The first age begins in the very infancy of society, as far back as historical tradition will allow, and comes down to the death of Galen, in the latter part of the second century of our era. This, the author calls the age of foundation. The second age, which may be termed that of transition, in which great principles were discussed, and symptomatology and prognosis "brought to a remarkable degree of perfection," extending to the date of the revival of letters in Europe, or to the beginning of the fifteenth century, offers few materials for a history of medicine. The art was stationary, if it did not actually retrograde. The third and last age, or that of renovation, in which the human mind seemed to awake, as from a long sleep, and displayed wonderful activity in every direction,—the sciences, fine arts, commerce, manufactures, social institutions, religious inquiries,—everything was changed or in process of change. The first age embraces four periods, viz., the *primitive* or *instinctive;* the *mystic* or *sacred;* the *philosophical;* and the *anatomical.* The second age has two periods: the Greek, and the Arabic. The seventh period, which begins the third age, is designated by M. Renouard as the *learned:* and the eighth period, which includes the seventeenth and eighteenth centuries, is named the *reforming.* The author has since given, in the shape of a supplement consisting of nine Letters, a critical review of the leading questions of the age which relate to medical doctrines and practice, and to medical heresies and absurdities. These Letters constitute the Appendix in the translation.

M. Gauthier, in his Inquiries into Temple Medicine, combats the opinion advanced by the animal magnetizers, that the priest-physicians had recourse to animal magnetism and to somnambulism in the treatment of the sick who were placed under their care. He describes the principal temples (*Asclepions* or *Asclepia*), dedicated to Esculapius, and the mode of practice adopted by the attending priests (*Asclepiadæ*); and also how dreams were interpreted into directions for the treatment of the sick, in these and other temples, in Greece and in Egypt. He contends that these institutions cannot be regarded in the light of hospitals, which he believes to be due to the influence of Christianity.

In the minds of many, the name of Hippocrates suggests, if not a mythical personage, at least a man whose history is mixed up with tradition; and of whom, at best, we have only a definite notion as the author or collector of some aphorisms. Accordingly, any person who might take it into his head to collect a certain number of herbs and roots to which tradition and popular belief ascribed curative powers, and who had watched the course of diseases, as a nurse, and a dispenser of his gathered simples, would be entitled to rank as the Hippocrates of his district and generation. Crude ideas like these might be excused among the ignorant and the unread; but they could hardly be expected to find a place in the brain of any medical man of common education—we might say, of common curiosity. If there be an excuse for such ignorance, it consists in the fact of the teachers in our medical schools, neglecting the history of medicine and the biographies of the great lights in the profession. The designating Hippocrates as "the Father of Medicine" has misled many into a belief that he was the first to treat of it with any method, and to make records of the symptoms of disease and their means of cure. A little attention to ancient history and a knowledge of the writings of Hippocrates himself suffice to dispel this illusion, and to show that there were physicians of eminence, schools of medicine, and clinical records, before the time of the Coan sage. The treatise which appears the first in the editions of his works, by his learned translators and commentators, Mr. Adams* and M. Littré,† is on "Ancient Medicine:" in it he finds fault with his predecessors, who had corrupted medicine by introducing hypotheses in explaining the causes of disease. Crotona, one of the great and flourishing cities of Magna Græcia which then included the greater part of southern Italy, was celebrated among other things for its medical school, a century before the birth of Hippocrates. To it belongs Democedes, a contemporary of Pythagoras, the latter of whom founded in that city his sect or order of hygienic philosophy.

Democedes, driven from home by the cruelty of his father, Calliphon, went first to Ægina, then to Athens, and afterwards to Samos, the tyrant of which island he cured of a dangerous disease. Taken prisoner by the Persians, he at first concealed his profession; but after awhile he was known, and was sent for by Darius, who suffered excessively from a dislocation of the

* The genuine works of Hippocrates, translated from the Greek, with a Preliminary Discourse and Annotations. 2 vols. London, 1849.

† Œuvres Completes D'Hippocrate. Traduction Nouvelle, Tome 1re, Paris, 1839.

foot, of which he was relieved by the skilful Crotonite. The queen, Atossa, who labored under cancer of the breast, had also recourse to him. The rewards and honors conferred on Democedes, at the Persian court, did not prevent him from feeling a great desire to see Greece once more. He obtained leave of absence, under the pretence of his intending to act as a spy; but in place of returning to Persia, he settled down, as we would term it, in Crotona, and married the daughter of his countryman, the celebrated Milo, of whose wonderful bodily strength and agility everybody has read. Iccus, of Tarentum, another of the great cities of Magna Græcia, paid particular attention to food, in its relation to the acquirement of the greatest bodily strength in gymnastic exercises.

In the Italian school, as that of Crotona has also been called, we must rank the philosopher, physician, poet, and patriot, Empedocles, of Agrigentum in Sicily, who arrested a pestilential disease, from which his native city had suffered greatly, by walling up a defile in the adjoining mountains, through which the wind brought noxious effluvia. He also rendered a similar service to Selinus, another city in Sicily, which had long been afflicted with malignant fevers, in consequence of the sluggish flow and almost stagnant state of the water of the river that surrounded its walls. This he accomplished by causing the streams of two neighboring rivers to be turned into the channel of the obstructed one, which was thus cleansed of accumulated filth; and both the town and its vicinity were restored to health. Empedocles was the author of two poems: one on *Nature,* of which a number of fragments are still preserved, and which contained some physiological explanations of the formation of animals; the other poem was entitled *A Medical Discourse.* He paid considerable attention to anatomy, and discovered the labyrinth of the ear, which he regarded as the part essentially concerned in hearing. He advanced certain physiological explanations on the causes of the difference in the generation of the two sexes, and on sleep, and death. Hippocrates, in the treatise before mentioned, refers to some of his doctrines. Acron, a contemporary and countryman of Empedocles, was a minute observer of phenomena, without reference to cause; and was adopted, a long time afterwards, by the Empirics, as the chief of their sect. He wrote a treatise on *Wholesome Food,* which has been lost. Better remembered and more celebrated than the preceding characters, just named, is Herodicus, of Leontinum in Sicily, and according to Plutarch, brother to Gorgias, the celebrated orator; while, by others, he is spoken of as a native of Selymbria. Herodicus at first presided over a gymnasium, which, like all institions of the kind in Greece before his time, was a school of bodily exercises of various kinds, to prepare the youth who resorted to them for becoming soldiers, or athletes. A witness of the strength obtained from these exercises by persons in health, it occurred to him that by the same means, under proper regulation, weakness and infirmity, induced by disease, might be remedied. He was, himself, of an infirm constitution, which probably served to quicken his attention to the subject, and prompted him to make the requisite experiments. He acquired great vogue for these *medical gymnastics*, of which he is usually considered the founder; but he must have allowed of abuses, to draw on himself the strictures of Hippocrates, who accuses him of carrying his exercises to a hurtful extreme, and especially in acute diseases. Plato objected to Herodicus, that the latter prolonged the life of valetudinarians beyond measure and expectation, in place of letting them die, and be entirely relieved of their ills. This opinion is worthy of Lycurgus himself, but is not one which the invalid parties themselves would feel disposed to adopt. We have yet another name to introduce, from the school of Crotona: it is that of Alcmæon, a disciple of Pythagoras, and who is described to have been the first to engage in practical anatomy, by the dissection of animals. Empedocles must also have enjoyed similar opportunities. We cannot dismiss the school of Crotona, without repeating the remark of the learned M. Littré, that Hippocrates drew freely from its doctrines; which, in this way, have exerted a powerful influence over the medical world.

In Greece itself, there were three sources of medical knowledge and means of medical instruction, anterior to the time of Hippocrates, viz., the Temples of Æsculapius, or Temples of Health; the Gymnasia; and the Schools of Philosophy, with all of which he was well acquainted, and from which he derived the materials and many of the doctrines of his own treatises. The abovementioned temples were called, as was before remarked, Asclepions or Asclepia, and the priests, who officiated in and presided over them, Asclepiadæ. These persons were at first the descendants of Esculapius, to whom, chiefly, the temples were dedicated. According to the Grecian story, this personage, who was one of the many sons of Jupiter, was so successful in curing diseases and preventing the approach of death, that grim Pluto made a formal complaint to his brother on Olympus, against such proceedings, which would, if allowed to go on, prevent any increase of the population of his lower empire. Jupiter, as a supporter of royal prerogative, listened to his brother's complaint, and killed Esculapius by a thunderbolt. No subsequent myth has made the followers of Esculapius liable to an accusation similar to that which cost him his life. Whether they have been deterred by his fate from saving so many lives, or have not been endowed with the same healing power, we cannot say.

The Asclepia combined temple with college, and, to a certain extent, hospital also. Their sites were eminently favorable to health, and suggestive of pleasurable sensations. They were usually on the summit of a hill or on the side of a mountain, sometimes not far from the sea-shore, and often in the vicinity of a medicinal, and even a thermal spring, and were sheltered by groves, amid which the senses were gladdened by flowers of variegated hues and agreeable odors. The newly-arrived sick underwent a preparatory process for receiving the gifts of health from the presiding deity, by previous ablutions, abstinence, and prayers. Religious ceremonies of an imposing character, often accompanied by music, were also practised. In the vicinity of the temple of Epidaurus, there was a large theatre for recitations and dramatic performances. The faith of the patients was strengthened by the priests reciting the wonderful cures performed by Esculapius; and pointing out, in confirmation, the votive tablets on the walls, and inscriptions on the columns of the temple. The patients, being thus prepared and predisposed, one might say, to be cured, were brought into the temple, in which they slept one or more nights, in order to be placed more directly under divine influence, or, more properly speaking, to have their imaginations affected by such sights and sounds as the priests might believe to be best adapted to the purpose. Faith and hope, powerful agents for the restoration of health, by their genial operation on the brain and nervous system generally, were thus brought into exercise; while, at the same time, the natural but too often neglected aids of pure and temperate regimen, with pleasing scenery, were enlisted in a subsequent period of the treatment. To this was added regular bathing, frictions, and gymnastic exercises, in the gymnasia adjoining the Asclepion. Riding on horseback was an Esculapian remedy, the practice of which is fully carried out at the present time by our brethren of the interior, and especially of the South and West; many of whom have never heard of the god of health, nor are aware that the ingenious fiction of the Greeks, which supposed the existence of centaurs, half man, half horse, arose from the sight of horse and rider, as if the two made one being, in the case of the inhabitants of Thessaly.

Active measures, covering, in fact, the list of ordinary therapeutical remedies, were not overlooked in the practice of the Asclepiadæ, or ministering priest-physicians; although the manner in which they were given was not in conformity with our modern Esculapian notions. The remedies were often selected on the faith of dreams, which were believed to be sent by Esculapius to the trusting invalid; but the interpretation of which, if they were allegorical or otherwise obscure, was made by the priests themselves. A wise discretion was, in some cases, absolutely necessary. Aristides, the orator, and therefore, as may be well understood, not Aristides the Just, was directed, in a dream, to lose a hundred and twenty ounces of blood, which was construed to mean that he ought to be freely bled. In our own day we might sometimes wish that there were persons of equal judgment as these priest-physicians, and invested with the like discretionary power to reduce to a prose and fact standard, the wonderful narratives, the waking dreams, to which some of our medical, not to say surgical, worthies are prone. On another occasion, a vicarious dreamer, one of the keepers of the temple, sorely puzzled, and at the same time naturally alarmed, poor Aristides. The dream was, that the bones and nerves of the orator were to be excised, as they were in a corrupt state. Happily, however, the god himself condescended to relieve the mind of the patient, by letting him know that recourse must be had to active means in order to bring about a change in his nerves and bones. It does not seem to have occurred to the credulous Aristides that the priests must have been indulging in a somewhat malicious satire on his own practice of exaggeration, and, sometimes, disguise of common facts, for the sake of producing a more lively impression on the minds of his auditors.

At times the dream was of a more pleasing character, and its requirements easily carried out; as in the case of Aspasia, the second, to whom Venus, under the form of a dove, appeared in a dream, and advised her to apply to a tumor on her chin dried rose-leaves, taken from the chaplets which had been given to her. Need it be added that this beautiful, what shall we call her—*Hetera*—was cured by the remedy!

The practices, enjoined on the faithful at the Asclepions, were not looked upon in the same favorable light by the more philosophical and better educated Greeks as they were by the multitude. Aristophanes, that great master of scoff and satire, went so far, in his comedy of Plutus, as to ridicule the tricks and mummeries of the priests, and their greediness of gain; and he caused the volatile and superstitious Athenians to laugh at exposures of the practice of a creed, philosophical doubts of the reasonableness of which made them condemn Socrates to the hemlock draught. Less exception could be found to the dreams suggested by Esculapius to the sick worshippers at his shrine, when they were recommended, as in the case of Marcus Aurelius, the emperor and philosopher, to take long walks, to ride on horseback, and to make use of the cold bath. Modern science has nothing to add to the various hygienic means suggested and put in practice in ancient temple medicine. Happy would it be for the general health at the present day, if we came up to the requirements, in this respect, of even the predecessors of Hippocrates. But, instead of carrying out the means of long-ascertained, if not of obvious, utility for the recovery of health, mummery without the excuse of superstition, and deception without the borrowed garb of hygiene, are substituted, under the names of mesmerism, animal magnetism, and last, and more impious than all, spiritual knockings, and table-turnings, and pretended revelations from the world of spirits, in the shape of the smallest and most silly gossip and twaddle, such as would fall short of the requirements of a kitchen tea-party. Priests of a purer faith and professed expounders of truly sublime doctrines are seen of late times, who, without actually practising, yield a too ready assent and attestation to these wretched devices, which can only be excused on the ground of insanity, preluded by this dominant hallucination. These persons seem entirely to forget that belief in the marvels thus set forth must destroy belief in true miracles; and, in the confusion of ideas thence resulting, reduce these latter to so many feats of mesmerism.

The mystic period of medicine in ancient Greece is revived in modern Christendom, in the shape of jugglery which sets all reason and experience, and the plainest rules of induction, at defiance. The present age affects a German spiritualism which is without faith, and, as a consequence, without religion; spiritualism only in its affecting to sublime matter, as, for instance, in homœopathic dilutions, which are beyond man's comprehension, and certainly beyond all possibility of any known or appreciable means of actual division or administration. The thing is impossible; but it is, notwithstanding, believed by many who, at the same time, disbelieve the mysteries of the Christian religion, because, save the mark, some do not understand them, while others think them explicable by the key of animal magnetism.

The Asclepiadæ of the different Esculapian and other health temples, for some were dedicated to other deities, as to Apollo, Venus, &c., formed colleges or corporations. The practice of medicine, at least of what might be called regular medicine, was entirely in their hands; and it seems to have been confined to certain sacerdotal families, as a kind of hereditary privilege. The instruction in them was begun in early life; it was almost entirely oral: Hippocrates was a member of one of these who were descended in a direct line, as it was alleged, from Esculapius. After a while, however, strangers were admitted for a fee, and taught the elements of medicine. Some of these became travelling or itinerant physicians, going from place to place, and from the court of one sovereign to that of another, to practise their profession. The strict Asclepiadæ must have allowed themselves eventually this privilege; for Hippocrates, who was in full standing in the order, by his birth, education, and associations, travelled in this way.

The most celebrated schools of medicine in temples, were those of Cnidos, Cos, Rhodes, and Cyrene. The two last-named were soon eclipsed by the fame of the others. Very different was the case with those of Cnidos and Cos, which may be said to have performed a conspicuous part in the diffusion of medical doctrines, and the formation of medical opinions. The first written work on medicine, so far as is known, emanated from the school of Cnidos: it is called *Cnidian Sentences*, and it furnished a theme for criticism by Hippocrates in one of his chief treatises (on *Regimen in Acute Diseases*). The most ancient of the Cnidian Asclepiadæ, whose name has come down to us, is Euryphon, a contemporary of, but older, than Hippocrates. He is generally spoken of as the author of the Cnidian Sentences, but, as Mr. Adams judiciously remarks, it is evident, from the language of Hippocrates in speaking of them, that they were written by more than one person. There were two editions of this work in the time of Hippocrates. Galen speaks of his having read it; but it is now entirely lost to us. Better fortune has attended the first books of Prorrhetics, and the Coan Prænotions, which, by Ermerius, whose opinion is adopted by M. Littré and Mr. Adams, are shown to have been written anterior to the time of Hippocrates. It may surprise some and amuse others of our readers, when we repeat, after M. Littré, that the school of Cos was engaged in the way of publishing later than that of Cnidos. It was customary for the sick, who had been benefited or cured by the treatment adopted in the Asclepions, to leave a few words expressive of their gratitude towards the tutelary divinity, and also describing the disease from which they had been freed, and the remedies by which they had been cured. These notes were inscribed on tablets suspended on the walls, or were written on the columns of the temple, and afterwards collected by the priests. The Coan Prænotions, introduced into the Hippocratic Collection, had probably been gathered in this way. The school of Cos attached great importance to a knowledge of the general symptoms, or those common to different diseases, that is, the symptoms which announced the efforts of nature; it also laid stress on those which mark the crises (probably the word belongs to this school), and the critical days. Such was the direction which the school of Cos had taken at the time that Hippocrates began his medical noviciate. The school of Cnidos, on the other hand, gave itself up to an observation and record of each separate symptom, and multiplied in a corresponding degree the varieties of disease. Thus, for instance, it spoke of seven diseases of the bile, twelve of the bladder, four of the kidneys, and four stranguries, besides three kinds of tetanus, four of jaundice, and three of phthisis. The Cnidian observers looked to the peculiarities of the body, instead of regarding the identity of the diathesis, as was done by Hippocrates. "In other words," says Mr. Adams,* "they split diseases into endless varieties, instead of attending to the essence or general nature of each. The system of Hippocrates then was founded on a rational prognosis, whereas that of the Cnidians was founded on mistaken principles of diagnosis." Dr. Watson, following M. Littré, sums up quite perspicuously the method of treatment adopted by the two schools, as follows: "In the management of

* Note to Regimen in Acute Diseases.

acute diseases, the Cnidians employed numerous remedies, and in other affections few. The school of Cos, though at times more heroic, especially in the use of the lancet and active purgatives, were in the habit of managing acute diseases by a restricted regimen; barley-water more or less diluted, hydromel and oxymel, being among their most frequent prescriptions. In the management of chronic diseases, they favored the medical gymnastics of Herodicus; whilst at Cnidos their diseases were managed principally by laxatives and a diet of milk, or of milk and water."

The second source of medical knowledge among the Greeks, in the times anterior to and succeeding those of Hippocrates, was the gymnasia and the labors of the superintendents of these institutions. In them all kinds of exercises were methodically taught, and observations made on the kinds of food best adapted to preserve health and strength; and rules were deduced for a suitable dietetic regimen, according to the difference in temperaments and ages. The frequency of dislocations and fractures among such large numbers as frequented the gymnasia must have imposed, almost of necessity, on the superintendents the duty of treating these accidents; and hence a school of practical surgery was opened, of which Hippocrates must have largely availed himself. Of the application of gymnastics to the cure of disease, and the origin of what may be called medical gymnastics, we have already spoken in connection with the name of Herodicus.

The third source of medical knowledge from which Hippocrates drew, was the school of the Philosophers, the most eminent of whom seem to have made medicine a subject of careful study and observation. They examined the organization of living bodies, chiefly of animals, and inquired into the origin of disease, for the prevention as well as the cure of which they relied greatly on dietetics. At the head of these was Pythagoras, who was born in the island of Samos, in the latter part of the sixth century before Christ, Anaximenes, Diogenes of Apollonius, and his successor, Empedocles, Heraclides, Democritus, and Anaxagoras, all of them living anterior to Hippocrates. Mention has been already made of Empedocles in connection with the Italian school of medicine. Anaximenes regarded air as the cause or principle of all things; an opinion advocated by Diogenes, who cultivated anatomy, and gave a description of the origin of the veins in the abdomen, and their termination in the heart, whence he traced them to the head. According to this philosopher, man's intelligence depends on the air which is diffused through the blood by all the veins of the body; he declares it to be necessary to the existence of all animals, and that even fishes breathe it in the water. We most devoutly wish that at the present time, in the middle of the nineteenth century, and somewhere about twenty-three hundred years since the time of Anaximenes and Diogenes, this doctrine of the necessity and importance of air found more ready credence and general practice than it receives when people are assembled for the purposes of worship, or of business, or amusement. It would seem as if to admit the free air of heaven on these occasions, was an impiety, or a piece of extravagance, or in bad taste. Anaxagoras, the preceptor of Pericles, is a philosopher whose doctrines have left their traces in the Hippocratic Collection. He attributed the cause of acute diseases to the bile; and we thus learn that the theory of the bile, as a cause of disease, was anterior to Hippocrates: yellow was also distinguished from black bile. Democritus was the most learned of the Greeks before Aristotle, and, like him, he was a man of universal knowledge. He directed his attention to anatomy, physiology, dietetics, epidemics, fever, and probably hydrophobia and convulsive diseases, and treated of all these subjects. Although his numerous works are lost, some medical terms which he employed are still in common use; such, for instance, as phagedenic ulcer. He was remarkable for the purity of his style; and Aristotle eulogizes in strong terms the profound scientific knowledge of Democritus. M. Littré gives a list of eight different treatises written by this eminent philosopher-physician. The loss of the one on plagues, or pestilential diseases, is particularly to be regretted.

Ancient medicine then, we repeat, was derived, during the fifth century before the Christian era, from three sources: the temple of Esculapius, or the Asclepia, the gymnasia, and the schools of philosophy. We learn, also, that Hippocrates was not the creator but the continuer of systematic medicine; and that he was preceded by many others in the treatment of diseases, both in the temples and elsewhere. He found to his hand a record of these diseases on tablets, which, although a votive offering by those who had been cured, were written out with care by the Asclepiadæ, and contained an account of the chief symptoms and the mode of treatment. He found also a collection of these notes, and publication of books (Cnidian Sentences, and Prorrhetics, and Coan Prænotions), and already evidences of two systems: the one, which consisted in noting all the symptoms, and in making almost as many separate diseases; the other, which ascertained what the symptoms had in common, as indicating the state of the powers of life and the course of the disease. Clinical records, systematic diagnosis and prognosis, were accessible to Hippocrates during his term of study, and furnished materials for his own subsequent observations and writings. Like his predecessors in the temple, he was at first, doubtless, a compiler of the cures on record, and he arranged them according to the method most approved at the time. Let us now indulge in a brief notice of this remarkable man.

Hippocrates was born some time between 450 and 460 years before our era, in the island of Cos. He enjoyed, very early, unusual advantages in prosecuting his studies, under the guidance of his father, Heraclides, in the Asclepion of Cos, where he had access to all the treasures of observation collected during many generations, and had an opportunity of assisting in the management of the sick. Out of the temple, in the gymnasia, he became familiar with the hygienic and curative effects of exercise and a regulated regimen, under such teachers as Herodicus; while, at the same time, he acquired surgical experience by assisting in the treatment of accidents—dislocations and fractures—occurring in the gymnastic exercises. Nor was the cultivation of his mind by the study of letters and philosophy neglected: in these he was instructed by Gorgias and Democritus. Thus prepared by early and suitably prolonged culture and training, and having obtained a medical rank as one of the Asclepiadæ, equivalent to what graduation confers in modern times, Hippocrates set out on his travels. He visited on this occasion not only Greece proper, but also Thrace and Thessaly, practising and probably teaching his art in those countries. He extended his observations to Asia Minor, then in part occupied by Greek colonies and cities, whose wealth and progress in philosophy and the arts were little inferior to their display in the parent states of Greece itself.

Incidents are related, as belonging to the life of Hippocrates, which we would fain persuade ourselves to have actually occurred; but, although often repeated, they lack historical proof. It must be confessed, indeed, that we have no authentic biography of the man, and that what little is known of him has been gleaned from occasional references by other writers. The only author entitled to confidence, who has written *ex professo* on the life of Hippocrates, is Eratosthenes, a learned astronomer of Alexandria, who lived two hundred years later; and he seems to have restricted himself to the genealogy of the great medical philosopher. The story told of the important part performed by Hippocrates in staying the great plague at Athens, is declared by M. Littré to be entirely destitute of foundation, on the ground of the silence of Thucydides in this historian's account of the disease, and also of the age of the physician himself, who was then only thirty-two years old. Mr. Adams, while he admits the failure of direct testimony, does not deny the probability of the services of Hippocrates having been put in requisition by the Athenians on the occasion of a subsequent outbreak of the plague. The same scepticism and denial are uttered by M. Littré in reference to the alleged invitation of Artaxerxes, king of Persia, to Hippocrates, to visit that kingdom, and to exercise his skill in abating the ravages of the plague, with which it was then afflicted. Mr. Adams, on his part, suggests the probability of a request of this kind being made, as Greek physicians had been before then at the Persian court, and it had become part of its policy to obtain an influence in Greece by gold, which it had in vain attempted by force of arms. We are also obliged to give up that pretty little piece of romance, of the detecting the love of Perdiccas of Macedonia for a concubine of his father, by Hippocrates noting the change of expression in the young prince when the object of his love entered the room in which he was detained by love fever. The same story is told of Erasistratus, with the addition, that he detected the cause of his patient's malady by the increased frequency of his pulse when the beloved one approached the sick man's couch.

Hippocrates was not only a keen and patient observer of diseases, and one of their earliest describers, but he was also a prominent physician and a medical philosopher—master of the literature of medicine, and, in fine, an accomplished and learned man. He was worthy to figure in the most brilliant period of Greece, we might almost add, the most so of any succeeding people in literature, philosophy, poetry, and the fine arts. Using the word contemporary in the large sense, of living in the same historical period, if not precisely in the same years, we may, with Mr. Adams, say of Hippocrates: "He had for his contemporaries, Pericles, the famous statesman; the poets Æschylus, Sophocles, Euripides, Aristophanes, and Pindar; the philosopher Socrates, with his distinguished disciples, Plato and Xenophon; the venerable father of history, Herodotus, and his young rival, Thucydides; the unrivalled statuary Phidias, with his illustrious pupils; and many other distinguished names, which have conferred immortal honor on the age in which they lived, and exalted the dignity of human nature."

Great difficulties beset the path of scholiasts and antiquarian critics in arranging the writings called the Hippocratic Collection, so as to determine which are really those of Hippocrates himself, and which are written by his family and immediate disciples, or by his successors at a later date. The greater part of the first volume of M. Littré, which is introductory to his translation of all the works of Hippocrates, with commentaries, is taken up with an investigation of these questions, into which it would be idle for us to pretend to follow him, in the space that could be occupied by only a few paragraphs. We would refer our readers, also, to an able inquiry on the same subject by Mr. Adams, in the "Preliminary Discourse" to his translation of the genuine works of Hippocrates; and, in the absence of those volumes, to a neat summary of the classification of the works composing the Hippocratic Collection, and of the character of each of those admitted to be genuine, by Dr. Watson, in his "Anniversary Discourse." It is very generally admitted that the following works are genuine, viz.: 1. On Ancient Medicine; 2. The Prognostics; 3. The Aphorisms; 4. The Epidemics, books first and third; 5. Regimen in Acute Diseases; 6. On Air, Water, and Places; 7. On the Articulations; 8. On Fractures; 9. On the Instruments of Reduction; 10. The Oath;

11. The Physician's Office; 12. Injuries of the Head; 13. The Law. The entire Hippocratic Collection is divided by M. Littré into eleven classes, placing in class first the thirteen treatises entitled as above, which are believed to be from the pen of Hippocrates. The works of the second class he attributes to Polybius, son-in-law of the old man of Cos. Two books placed in the third class are believed to be more ancient than the genuine writings. These are the Coan Prænotions and the first book of Prorrhetics, to which reference was made in a preceding page.

Hippocrates gave what is most desired, the natural history of diseases. He laid great and deserved stress on prognosis, which implies not merely predicting the result, but a careful appreciation of the previous and present condition of the patient. Throughout, he advocates, and for the most part successfully preserves, the inductive method. His descriptions of disease bear proof of their being the result of his own observations, and of his having well reflected on those of other writers. His rules of practice are based on experience, but a rational not an empirical experience. Among the distinguishing traits of Hippocrates, are an exalted idea of medicine, its extent, its aim, and its difficulties; a great regard for medical dignity, and a lively feeling of the duties and obligations of his profession; a deep aversion against those who in any way compromised it, either by quackery or immorality; and, in fine, his unceasing solicitude for the cure, or, failing in this, the relief of the sick. The most philosophical of the works of Hippocrates, that which evinces extended observation, travel, and study, is on "Airs, Waters, and Places." In it, he inquires into the effects of the seasons, winds, and various kinds of water, localities, nature of the soil, and modes of life and exercise upon health, and the necessity of a physician making himself acquainted with all these matters. He next points out the influence of climates, and the diseases depending on differences in them. He compares the people of Europe, and especially of Greece, with those of Asia, and shows how the uniformity of the climate and the fertility of the soil in the latter, induce a monotonous course of life and thought, and disinclination to exertion in the inhabitants, who, in consequence, give themselves up to indolence and love of ease; whereas the Europeans, living on a poorer soil and in a climate of frequent vicissitudes, are compelled, for self-protection, to exert themselves in various ways; and thus they acquire habits of self-reliance, and display greater courage. Hellenic pride and consciousness of superior advantages, speak through the medical philosopher, when he tells us: "On this account, the inhabitants of Europe are more warlike than the Asiatics; and, also, owing to their institutions, because they are not governed by kings, like the latter; for, where men are governed by kings, there they must be very cowardly, as I have stated before; for their souls are enslaved, and they will not readily or willingly undergo dangers, in order to promote the power of another; but those that are free, undertake dangers on their own account, and not for the sake of others; they court hazard, and go out to meet it; for they, themselves, bear off the rewards of victory; and thus these institutions contribute not a little to their courage." Ethno-climatic and political teachings of this nature ought to find willing disciples among the people of the United States.

The books of "Prognostics" may not unaptly be called the Commentary of Hippocrates on the Coacæ Prænotiones; and it is regarded by competent critics as the oldest of all the works which the "Father of Medicine" has left to us. The rules of Prognosis, laid down in this treatise, are manifestly those by which the author is regulated in his other works, and more especially in the Epidemics and Aphorisms. In the treatise "On Regimen in Acute Diseases," two very important questions, as remarked by Mr. Adams, are discussed: first, a nosological question, regarding the proper distinction of diseases from one another; and, secondly, a therapeutical, respecting the rules by which the regimen in acute diseases ought to be regulated.

We are unable, now, to give even a slight outline of the subjects treated of, in the different treatises of Hippocrates; but must reserve for a future occasion the discharge of this pleasing duty. We shall, perhaps, be able to show the resemblance between the fevers (causus) described by Hippocrates, in the first and third Books of Epidemics, and the bilious remittent fever, which is met with at the present day in the Morea; and the like of which is common enough in Algeria, and in portions of our own country. If these subjects are investigated, the doctrines of Hippocrates respecting the humors, and critical days, will come up as collateral topics. We can now allude only to one more of the medical treatises of the great teacher, viz., the "Aphorisms," which, as has been justly said by the English editor and translator, "whether we regard the value of the subject-matters of which it is composed, or the influence which they have exerted on professional practice during a long lapse of ages, may be confidently pronounced to be one of the most remarkable works in the whole compass of medical literature." This work may be regarded as a summary view of the chief results of the preceding inquiries of the author.

The surgical writings of Hippocrates commend themselves to attention even in this age of progress; not merely as descriptive of the means had recourse to in fractures and dislocations twenty-two centuries ago, but as furnishing to the modern surgeon practical knowledge, by the clear and minute details of the injury, and the use of apparatus, and other methods of treating it. On the accidents which befall the elbow joint, "it will be admitted, even at the present day, that his information on the subject was almost complete. He was a bold operator: he trepanned the skull, and opened the chest in empyema and in hydrothorax. His views regarding club-foot were the soundest of all who had written on the subject, until the recent times of Stromeyer and Delpech.

Hippocrates passed the latter years of his life at Larissa, in Thessaly; and there ended his days, at the age of eighty-five years, according to some accounts, and of ninety, and even one hundred and four, and one hundred and nine, according to others. He was buried between Gyrton and Larissa, in a spot where subsequent writers asserted that his tomb was still to be seen. The Greeks, with their ever-inventive minds, took pleasure in telling, that long afterwards his tomb was the foundation of a bee-hive, the honey from which had the property of curing aphthæ of children. The lustre thrown over the school of Cos by Hippocrates was continued by his own immediate family, in the persons of his two sons, Thessalus and Draco, and his still more eminent son-in-law, Polybius. After these come Diocles, who was called by the Athenians, the second Hippocrates, and Praxagoras, of Cos, who was the last of the family.

The most celebrated among the philosophers, who directed his attention and studies to medicine, and who became imbued with the doctrines of Hippocrates, was Aristotle. Left to his own guidance, by the early loss of his father, Aristotle did what many young men do in every age of the world,—he spent his patrimony in dissipation, and became a soldier, like the Scotch Buchanan, and bluff Ben Jonson, in later times. Returning to his early studies—philosophy and medicine—he gave himself up to them with uncommon ardor and perseverance; and he became one of the most devoted disciples of Plato, who was then the great teacher in Athens, and to whom the youth of genius resorted from all parts of Greece. Aristotle persevered under the discouragement of straitened means, if not actual poverty,—a fact which, of itself, would entitle him to a place in the Esculapian brotherhood. It is said, that in order to procure for himself the means of subsistence, he kept a shop for the disposal of herbs; and, at the same time, he gave advice to the purchasers of his medicines, according to the usage of the pharmacopolists of that day. An humble beginning this, for the preceptor and friend of Alexander the Great, who placed at his disposal all that power and wealth could procure, of the rarest animals and plants, and other objects of natural history, in the various countries conquered from the Persian king. Aristotle belongs to the history of medicine in various ways: first, by his birth, he being the son of a physician; secondly, by his having, as it has been alleged, practised medicine during some years of his life; thirdly, by his researches in comparative anatomy and physiology; and, finally, by his philosophical doctrines, which exerted, for so long a period, a paramount influence over the mode of teaching all the branches of human knowledge, in the greater number of which he was himself eminent. The division of the functions of the living body, made by Aristotle, is nearly the same as that adopted by Bichat and many other modern physiologists, viz., into the nutritive or vegetable, the sensitive, the motor, and the intellectual. He gives a much more minute and accurate description of the brain, than his predecessors. Nor must it be forgotten, to the honor of Aristotle, that his arrangement or classification in comparative anatomy is that which has been adopted twenty centuries later, by the eminent Cuvier. Aristotle, in his "Politics," in drawing the traits of the European and Asiatic character, borrowed freely from the treatise of Hippocrates, on "Airs, Waters, and Places," as Montesquieu and Cabanis have, in later times. His great merits, as a critic and profound writer on politics and government, do not come within the field of our present inquiries.

There is no great name, nothing truly original or pointedly useful in the history of medicine, that need detain us from a notice of the schools of Pergamus and Alexandria, and the immense libraries and museums by which these cities became, for centuries, the abodes of science and literature, of which medicine constituted a leading share. The happy idea seems to have occurred to both Eumenes, king of Pergamus and Mæsia, and to Ptolemy Soter, king of Egypt, to found public libraries—the former at Pergamus, the latter at Alexandria. Some interesting particulars on this subject are given by M. Renouard, in his history of what he calls the anatomical period, or that between the foundation of the library of Alexandria and the death of Galen—from 320 B. C. to A. D. 200. In the time of the great physician just named, it was enough to have studied in Alexandria, or even to have remained there for a season, to be looked on as a physician. One powerfully contributing cause to the success of Græco-Egyptian medicine was the liberty given to its leaders to engage in the dissection of human bodies, in which some of the Ptolemies themselves took part *en amateur*. This privilege was abrogated by the conquest of the Romans, who, notwithstanding their delight at seeing blood shed, either on the field of battle or in the combats of the gladiators, looked on contact with a dead body as a horrible profanation. Their coming was productive of another calamity—the burning of the great library, by Julius Cæsar, or, at least, by the flames extending from his fleet to the library. The great names of the Alexandrian school, in its first and most flourishing period, under the Ptolemies, were, in connection with anatomy and physiology, Herophilus and Erasistratus, who carried these subjects much further than had ever been done previously. The former was the first to direct attention to the pulse as an index to the state of the body in health and disease; the latter was distinguished for his skill in the practice of medicine Surgery also engaged their attention. Although they are classed under certain medical sects, both Aretæus and Cælius Aurelianus, who lived in the second century after our era, may be regarded as of this school: their treatises, describing both acute and chronic diseases, constitute, it has been said, one of the most precious and useful monuments of ancient medicine. To the former must, however, be awarded the superiority, both in the description and semeiotics of disease, and in purity of style. There is, perhaps, Dr. Watson remarks, no modern writer to whom Aretæus can be more aptly compared than to Heberden. By some he has even been placed above Hippocrates for truth and accuracy of description. His surgical, in the opinion of the same writer, is in keeping with his medical ability. He was the first, as far as Dr. W. remembers, to use the trephine for the cure of epilepsy. Cælius Aurelianus wrote in bad Latin, and is spoken of as a follower and copier of Soranus; but yet, the eight books constituting his work abound in valuable descriptions, not only of diseases, but, still more, of their treatment. He may be considered as the ablest exponent of the methodic doctrines.

Next to Hippocrates, no name in ancient medicine looms out in such bold and bright relief as that of Claudius Galen. If in it Hippocrates be the first great historical personage, Galen is unquestionably the second. No other of equal celebrity intervenes to break the invariable connection between the sage of Cos and the profound scholar of Pergamus, in all our discourses on the progress of medicine and the influence of medical doctrines. Not more inseparably connected are the names of Socrates and Plato in philosophy, than those of Hippocrates and Galen in medicine. There is, also, a resemblance between the two philosophers and the two physicians, in the fact that, as Socrates is mainly indebted for his enduring fame to the eloquent discourses and "divine dialogues" of Plato, so is Hippocrates most indebted for his diffused and subsequent reputation to the learned commentaries and expositions of Galen.

Galen has every title to our respect for his vast learning and critical acumen, his extensive and unwearied inquiries into every department of medicine, his unbounded zeal for its improvement, and, Pagan though he was, for his fervent piety. Where, in the nineteenth century of Christianity, shall we read a finer burst of religious feeling by a Christian physician than when he tells us (*De Usû Partium*, Lib. III, Cap. 14) that, in writing these physiological treatises he was composing "a true and real hymn to that awful Being who made us all; and in my opinion true religion consists not so much in costly sacrifices and fragrant perfumes, offered upon his altars, as in a thorough conviction impressed upon our own minds, and an endeavor to produce a similar impression upon the minds of others, of his unerring wisdom, his resistless power, and his diffusive goodness;" and so in the same strain of appreciation of the wisdom of the Deity, and of adaptation in all his created works.

Galen was born A.D. 131, in Pergamus, in Mæsia, Asia Minor. His native city was early celebrated for a temple dedicated to Esculapius, and still more, in after times, for its school of medicine and its library, second only to those of Alexandria itself. From his father, Nicon, an able mathematician and architect, he received a careful education, with a view to his studying medicine; although he himself tells us that he felt called to do so by an injunction of Apollo, conveyed in two successive dreams. Of his teacher, progress in his studies, travels in distant countries, his surgical appointment on his return to dress the wounds of the combatants in the circus, and his quitting his native city for Rome, we would speak in detail, were room allowed us. A pleasing sketch of the life and merits of this celebrated man is found in Dr. Watson's Discourse; and critical notices of his works are given by M. Renouard. The success of Galen in Rome excited the envy, and his naturally haughty deportment drew on himself the hatred, of the physicians of Rome; and hence his leaving that city and again travelling. He was greatly esteemed by Marcus Aurelius, and enjoyed the confidence of Commodus, and Septimius Severus, the successors of this emperor. He seems to have stood high in the good opinion of the Empress Faustina, and, as a consequence, he became the favorite of the ladies of the court, and the fashionable physician in Rome. He retired in the decline of his days to Pergamus, where he died, according to the most approved statement, in the seventy-first year of his age; but other accounts would make it appear that he survived one hundred and forty years.

There was no branch, scarcely a topic in medical science, on which Galen did not write, and write with signal ability. In his belief of coction and critical days he is entirely in accordance with the school of Hippocrates. He divides the action of medicine into primary and consecutive,—a division and distinction too often lost sight of. Taking his treatise (*De Locis Affectis*) in connection with his writings on anatomy and physiology, they constitute imperishable titles of honor to the physician of Pergamus, and justify, or at least excuse, the extravagant admiration manifested for him during so many successive ages.

His *Ars Medica* was for centuries the text-book upon which the students of Salernum and other schools of the middle ages were examined before receiving permission to practise. Galen was famous for his skill in diagnosis, between which and prognosis he drew lines of distinction which are far from being appreciated as they ought to be by modern physicians, and perhaps less by those of our own than of any former time. It seems to be forgotten, that symptomatology and semeiology or semeiotics are parts of pathology, which latter is now, not only by mere morbid anatomists, but, also, by authors of systems of medicine, almost restricted to a study or observation of diseased structure, only detected after death.

On the fruitful subject of Hygiene, which, in its large and comprehensive sense, is so much neglected, Galen wrote at great length, as well in original treatises as in his commentaries on some of the books of Hippocrates. The doctrine of temperaments, if not entirely original with Galen, was first presented in a shape which, with modifications, has obtained currency in the schools down to the present time. His two most noted works on hygiene are, On the Preservation of Health (*De Sanitate Tuendâ*), and on the "Properties of Aliments." He dwells more than any of his predecessors on the hygienic rules applicable to infancy and old age; and he points out also the influence of habits, and was the first to feel that a regulation of the passions ought to be a part of hygiene.

This truly encyclopediacal writer has rendered invaluable services to the history of medicine by preserving for us the opinions of a large number of physicians whose works have perished, and especially of those of the leaders of the different medical sects. Following the division made by our venerable confrere, Dr. John Redman Coxe, in his summary outline of the works of Galen, which is appended to his abridgment of the treatises of Hippocrates, we meet with, 1. General Introductory; 2. Such as Appertains to Anatomy; 3. To Physiology; 4. To Hygiene, including the Practice of Physic and Surgery; 5. To Materia Medica and Alimentaria; 6. To Philosophy and Metaphysics; 7. To Miscellaneous Subjects. The writings of Galen are said to consist of seven hundred treatises or books; some of them, it is true, not more than what in the present age would be called pamphlets. Other persons, again, represent his contributions to medical science to be equal to five hundred volumes, and to other subjects the same number.

In continuing the outline history of the schools of Alexandria and Pergamus, we find ourselves in advance of the Roman school, as regards chronological order. The first person of note in it was, however, an Asiatic Greek, Asclepiades, born in Bithynia, and educated in the school of Alexandria. He was at first a teacher of rhetoric in Rome, and afterwards took to medicine, in the practice of which he acquired great vogue, and won for himself the friendship of Cicero, who speaks of him not only for his skill in physic, but also for his refined eloquence; a gift turned by him to good account in enforcing his principles of medicine, which consisted chiefly in the observance of diet and other hygienic measures. Among these he laid great stress on frictions of the skin, in connection, we must suppose, with the use of the warm bath, then becoming general in Rome.

Asclepiades was the preceptor of Themison, chief of the Methodists,—

a medical sect thus called, which we hope none of our readers will confound with Christian Methodism. Celsus must be regarded as the first physician of the Roman School who was a native. Aurelius Cornelius Celsus has been called, but it seems to us not very appositely, the Latin Hippocrates, unless veneration for him, the wise old man, of Cos, and the large borrowing from his writings, and the adoption of his opinions, invest Celsus with a filial character, and give him a kind of claim on the family name. He was eminent among his contemporaries for his varied learning and accomplishments; having written on rhetoric, philosophy, architecture, husbandry, and the military art. All these works are lost; but, happily for our science, both in its practice and history, his eight books on medicine have come down to us. They form a concise, yet clear and methodical abridgment of ancient medicine, from the time of Hippocrates to his own, which was in the first century of our era. For our inability to lay before our readers, at this time, the prominent features of the opinions and the works of Celsus, we find consolation in knowing how well this duty has been performed by Dr. Watson, whose sketch of the whole Roman School is one of the freshest parts of his volume, as less in the line of the common if not routine history of medicine. In this part of his Discourse, Dr. Watson introduces the prominent traits of doctrine and creed of the Rationalists, Empirics, and Methodists,—a theme discussed also by M. Renouard, somewhat more at length, but not, we think, with equal felicity. Coming within this period, and in the first century of our era, we meet with the name of Dioscorides, of Anazarba, who has left a Materia Medica, in five books; being the most complete treatise up to his own time, and the one to which we must ever remain mainly indebted for what is known of the views and practices of the ancients on this subject. The other two writers who have contributed to the same object are Pliny the elder, and Galen.

Under the head of the "Age of Transition," and a specified time, which he calls the "Greek Period," M. Renouard carries us along from the epoch of the death of Galen, A. D. 201, to the destruction of the Alexandrian Library by the Saracens, A. D. 640. During this period there offers to our serious study only the life and works of four Greek physicians, who had all studied in the School of Alexandria, which continued to maintain its reputation down to the invasion of Egypt by its Arabian conquerors. Although these writers were chiefly compilers of the works of Galen and of other authors preceding their own time, they have nevertheless, as well remarked by our French historian, rendered essential service by presenting medical science in an abridged and more commodious form, while, at the same time, they enriched it by some contributions of their own; and perhaps they did better service still in keeping up the light which might otherwise have been allowed to be extinguished by their apathetic contemporaries. First of these is Oribasius, of Pergamus, who is scarcely inferior in learning and attainments to his world and all time renowned countryman, Galen. He was the friend and trusted counsellor of the Emperor Julian, the Apostate, at whose instance he undertook an abridgment of the works of Galen, and afterwards a compilation of everything of value in those of his predecessors. This task he accomplished in seventy books, of which seventeen only are extant. He afterwards prepared an abridgment of his large work for the use of his son, which was divided into nineteen books, and which has come down to us entire, as well as another compendium on the preparation of remedies and the cure of diseases. As a French translation of Oribasius, by M. Daremberg, is in progress in Paris, we may be induced to bring him before our readers in a more satisfactory and instructive manner than we can now by the mere introduction of his name, and the mention of the character of his works. Oribasius may be regarded as the last of the Pagan writers in medicine, as Ætius is the first Christian one of any note. This latter, a native of Amida in Lydia, lived towards the end of the fifth century, and beginning of the sixth : he is entitled to our attention, and, we might add, to our gratitude, on the same grounds with Oribasius, as the author of a valuable compilation of the works of the older writers, interspersed with his own original observations. His work is divided into four books, each consisting of four discourses. Alexander Trallianus, so called from his native place, Tralles, in Mesopotamia, after travelling through most of the countries which once made up the Roman Empire in the plenitude of its dominion, fixed his residence in Rome, where he soon acquired a brilliant reputation. Having reached an advanced age, and feeling himself no longer able to encounter the fatigues of practice, he set about composing a treatise, in twelve books, on diseases in which surgical aid is not demanded. Like Aretæus, he restricted his descriptions to a small number of diseases, some sixty in number, as those of which they had acquired personal knowledge. In point of originality, Alexander ranks next to Hippocrates and Aretæus. Paulus Ægineta, or Paul of Ægina, designated after the island in which he was born, is the last Greek writer to engage our attention, and with him we conclude this rapid historical sketch of ancient medicine. He is supposed to have been a professor in Alexandria about the period of its capture by the Saracens, and the destruction of its library, which followed that event. He at first did not pretend to do more than to give an abridgment of Oribasius; but, in the progress of his work, Paulus went beyond his original intention, and drew from many other sources, as well as from his own experience, especially in matters of surgery and obstetrics. The English reader has now access to this most valuable compilation of ancient medicine in the translation, with pertinent annotations, by Mr. Francis Adams, which has been brought out by the Sydenham Society of London.

We cannot separate from Dr. Watson without expressing our strong desire to see a somewhat similar account to that furnished in his "Anniversary Discourse" "of the Profession among the Arabs of the East and West, among the Byzantine and Latin Schools, and the Monastic Medical Institutions of the Middle Ages," for which he has, we learn, the materials on hand. It would give us much pleasure to be able, once more, to avail ourselves of his company and instructive remarks, at the same time that we should be drawing from the stores contained in the second and larger part of M. Renouard's History, which the present is not the opportune time for introducing to our readers.

